# More Than Meets the Eye: Isolated Bilateral Abducens Nerve Palsy as the Initial Presentation of Multiple Sclerosis

**DOI:** 10.7759/cureus.27176

**Published:** 2022-07-23

**Authors:** Pius E Ojemolon, Rebecca E Enejo, Endurance O Evbayekha, Livio U Ituah, Hafeez Shaka

**Affiliations:** 1 Internal Medicine, John H. Stroger, Jr. Hospital of Cook County, Chicago, USA; 2 Department of Surgery, Federal Medical Centre, Lokoja, Lokoja, NGA; 3 Internal Medicine, St. Luke’s Hospital, St. Louis, USA; 4 Department of Anatomical Sciences, St. George’s University School of Medicine, St. George’s, GRD

**Keywords:** myelin oligodendrocyte glycoprotein-associated disorders, idiopathic intracranial hypertension, cranial nerve palsies, neuromyelitis optica spectrum disorder, abducens palsy, demyelination, multiple sclerosis

## Abstract

Multiple sclerosis (MA) is a chronic demyelinating disease of the central nervous system. Although the initial presentation of MS is widely variable, only rarely does it present with isolated bilateral cranial nerve involvement. With this article, we report a case of MS initially presenting as a clinically isolated syndrome of bilateral abducens nerve palsy.

## Introduction

Multiple sclerosis (MS) is a chronic demyelinating disease of the central nervous system (CNS) that has a widely variable presentation [1]. About 2.8 million people are estimated to live with MS worldwide, with the prevalence noted to have increased globally since 2013 [2]. In the United States, estimates of the prevalence of MS range from 400,000 to 900,000 persons. Females are two to three times as likely as males to have MS [1-3].

Isolated cranial neuropathies are rare in MS but are more likely to occur as the initial presenting symptoms than as part of MS relapses. The abducens nerve is the third most commonly affected cranial nerve in MS (after the trigeminal and facial nerves) [2,4]. Most cases of abducens nerve palsy in MS are unilateral and associated with other cranial nerve deficits and brainstem lesions [5].

In this article, we present a case of acute-onset bilateral abducens nerve palsy, with workup eventually revealing a diagnosis of MS.

## Case presentation

An African American lady in her early 30s with no known medical history presented to the emergency department on the advice of her optometrist with double-vision for two weeks. Her symptom was sudden in onset and initially associated with nausea which resolved after a week. Her vision worsened when she looked at distant objects, or to the sides, and improved when she occluded either eye. However, she denied headaches, visual loss, transient visual obscurations, blurred vision, pain with eye movements, impairment of color vision, droopiness of the eyelids, slurred speech, dysphagia, tinnitus, or weakness and numbness of her face or extremities. A review of the systems was negative for fevers, chills, neck stiffness, recent changes in weight, head trauma, loss of consciousness, or seizures. No prior history of visual or neurologic problems was reported. There were no recent respiratory infections, and there was no family history of neurologic conditions.

Her body mass index was 54 kg/m^2^ (normal range: 18.5-24.9 kg/m^2^). Eye examination revealed left eye esotropia with bilateral abduction deficit worse on the left side without pain on ocular movements. No nystagmus was demonstrated. Her pupils were 3 mm in diameter, and direct and consensual light reflexes were intact bilaterally. Her optic discs were non-edematous. Vibration sensation was diminished in both feet, but her plantar reflexes were down-going bilaterally. The rest of her physical examination was unremarkable.

Her basal metabolic panel and complete blood count were unremarkable. Syphilis enzyme immunoassay, human immunodeficiency virus screening, anti-nuclear antibody, and hepatitis B and C serologies were negative. Other pertinent laboratory results are presented in Table 1.

**Table 1:** Pertinent laboratory results.

Investigation	Result	Reference range
Erythrocyte sedimentation rate	18 mm/hour	0–23 mm/hour
Serum C-reactive protein	1.24 mg/dL	0–0.5 mg/dL
Total hemolytic complement	>60 units/mL	31–60 units/mL
Serum vitamin B12 level	205 pg/mL	200–900 pg/mL
Serum methylmalonic acid level	129 nmol/L	87–318 nmol/L
Serum folate level	6.22 ng/mL	5.9–24.8 ng/mL
Serum angiotensin-converting enzyme level	48 units/L	9–67 units/L
Serum soluble interleukin-2 receptor-α	1,575 pg/mL	532–1,891 pg/mL

**Figure 1:**
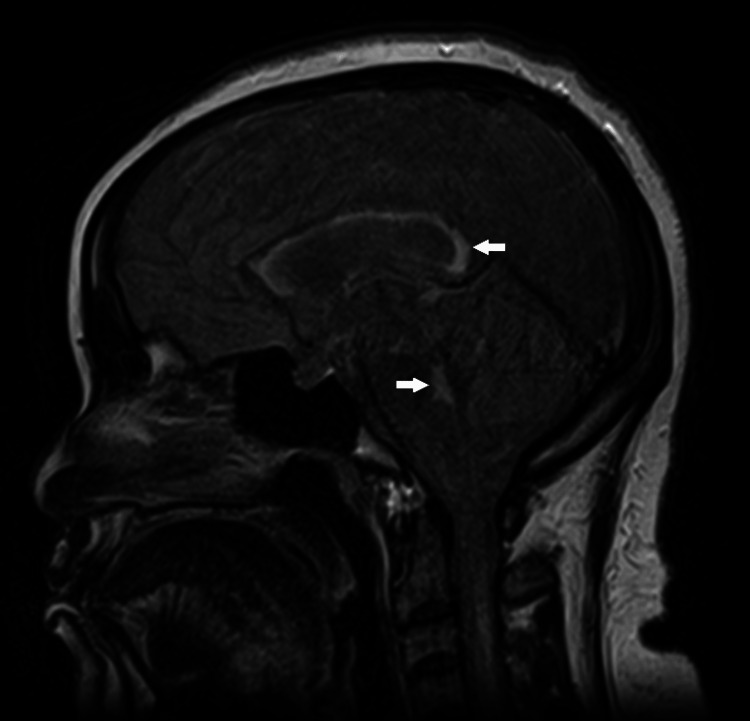
Sagittal MRI showing enhancements (white arrows) in the periventricular and dorsal pontine regions. MRI: magnetic resonance imaging

**Figure 2:**
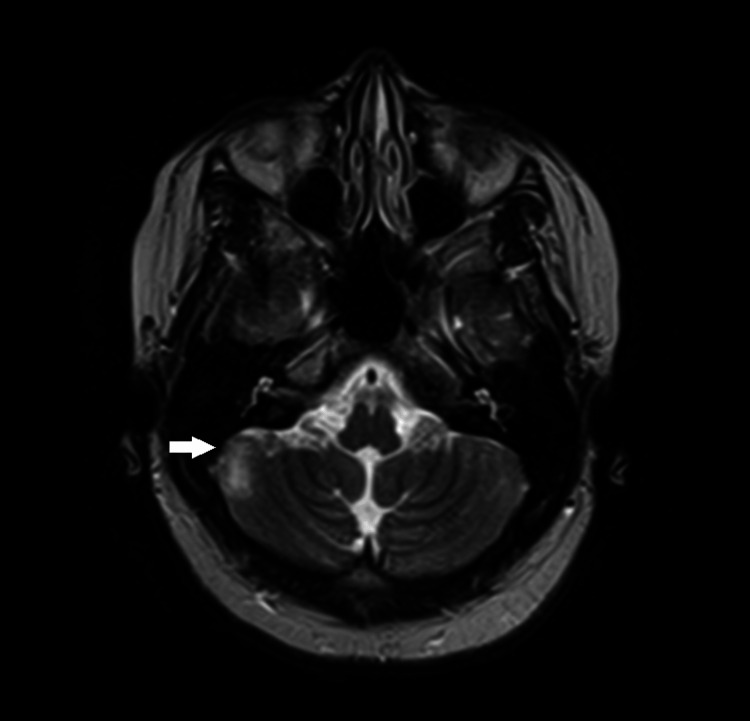
Axial MRI showing FLAIR signal hyperintensity (white arrow) in the right aspect of the cerebellum. MRI: magnetic resonance imaging; FLAIR: fluid-attenuated inversion recovery

Non-contrast computed tomography of the head showed symmetric enlargement of the lateral and third ventricles, greater than expected for age-related ex vacuo dilatation, and low attenuation of the white matter around the margins of the lateral ventricles. Magnetic resonance imaging (MRI) of the brain demonstrated diffuse volume loss, greater than expected for the patient’s age, with ventriculomegaly appropriate for the degree of atrophy. Diffuse fluid-attenuated inversion recovery (FLAIR) signal hyperintensities were seen in the periventricular white matter, callosal septal interface, right aspect of the cerebellum, and dorsal aspect of the pons (Figures 1, 2).

**Figure 3:**
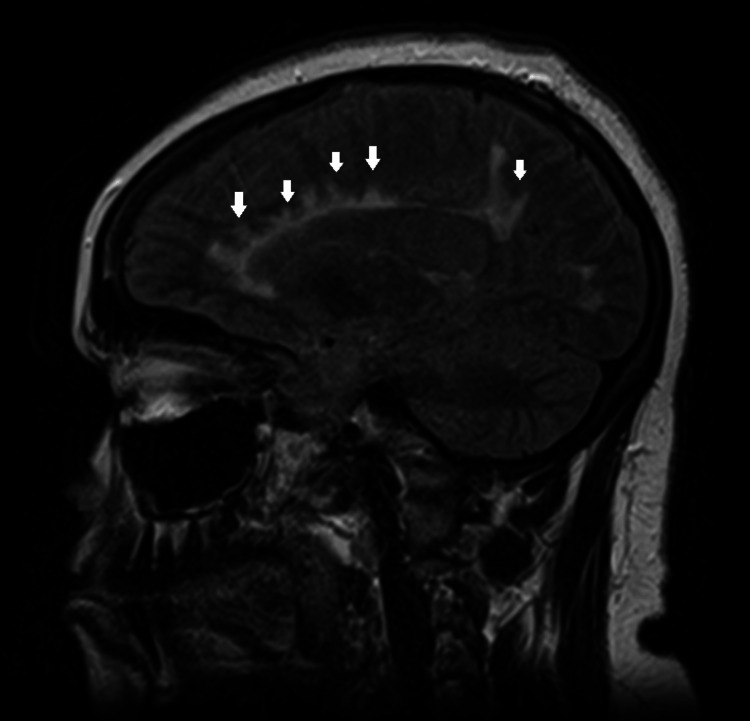
Sagittal MRI showing periventricular enhancement with radiations (white arrows) perpendicular to the body of the lateral ventricle (Dawson’s fingers). MRI: magnetic resonance imaging

Radiations were emanating from the periventricular enhancements perpendicular to the body of the lateral ventricle (Figure 3). T1 black holes were also noted in the periventricular white matter. The optic chiasm was grossly unremarkable and the sella turcica was empty. Magnetic resonance angiography of the head showed patent anterior and posterior circulation with normal flow-related loss of signal in the dural venous sinuses and normal appearance of the cavernous sinus.

The constellation of imaging findings was most suggestive of MS with a moderate burden of demyelination, but given her age, body habitus, and empty sella on MRI, a lumbar puncture (LP) was ordered to rule out idiopathic intracranial hypertension (IIH). She underwent fluoroscopic-guided LP. Her cerebrospinal fluid (CSF) was clear and colorless, with an opening pressure of 15 cmH_2_O, which ruled out IIH. CSF glucose was 58 mg/dL (normal = 50-70 mg/dL), protein was 15 mg/dL (normal = 20-50 mg/dL), with less than one white blood cell per µL (normal = 0-5) and two red blood cells per µL.

She was commenced on high-dose intravenous pulse steroid therapy with methylprednisolone 500 mg q12 hours with rapid improvement in her diplopia. CSF culture eventually yielded no growth, while CSF oligoclonal bands were positive. Serum anti-myelin oligodendrocyte glycoprotein and anti-aquaporin 4 antibodies were negative. She was discharged home after a total of six doses of methylprednisolone and on an oral steroid taper to continue as an outpatient follow-up at the neurology clinic. She is being planned for commencement of ocrelizumab therapy.

## Discussion

MS can affect any area of the CNS and ranges in presentation from asymptomatic (if non-eloquent parts of the CNS are affected) to severely disabling disease with cognitive impairment and decreased quality of life [6]. The periventricular white matter is the most frequently affected site. Brainstem involvement is common in MS, and multiple areas of the brainstem are often affected by the demyelination process; hence, isolated cranial nerve palsies are rare [7].

Abducens nerve palsy can arise due to a nuclear or fascicular lesion of the sixth cranial nerve. Among isolated cranial nerve palsies in MS, the trigeminal nerve (4.8%) and facial nerve (3.7%) are more frequently involved than the abducens nerve (1%) [5,7]. Given their close relationship in the pons, all three cranial nerves can be affected simultaneously. However, subtle facial numbness or weakness may be unappreciated and are therefore underreported [8]. In contrast, abducens nerve palsy (unilateral or bilateral) is the most commonly reported cranial neuropathy in cases of IIH, likely a non-localizing effect of raised intracranial pressure on the abducens nerve with its long intracranial course before reaching the orbit [9,10]. Moreover, IIH tends to affect a similar epidemiological group to MS (young and middle-aged females) [9]; hence, in presentations such as our case, it is expedient to rule out IIH in the workup of such a clinically isolated syndrome suspicious for MS [10].

Patients with isolated bilateral abducens palsy typically present with distressing diplopia upon horizontal gaze and may report improvement of symptoms with occlusion of either eye, as in our patient. Physical examination reveals slow abduction of the eyes and failure of either eye to cross the midline on abduction. Eye movements in other directions are unaffected unless there is a lesion of the oculomotor or trochlear nerve in the midbrain [11].

In patients presenting with isolated abducens nerve palsies, MS and IIH must be considered in the differential diagnosis. When MS is suspected, mimickers such as neuromyelitis optica spectrum disorders and myelin oligodendrocyte glycoprotein antibody-associated disease should be ruled out by serological workup (testing for antibodies against aquaporin-4 and myelin oligodendrocyte glycoprotein, respectively). Most cases of abducens nerve palsy occur in patients with raised intracranial pressure due to head trauma, tumors (particularly of the skull base), aneurysms, and ischemic strokes. In patients younger than 50 years, infectious causes (Lyme disease, meningitis, syphilis), sarcoidosis, autoimmune vasculitis, and Duane syndrome should also be considered. Among older patients, small-vessel vascular disease, giant cell arteritis, and diabetic neuropathy should be considered as well. Other possible etiologies include cavernous sinus thrombosis, Wernicke-Korsakoff syndrome, and Guillain-Barre syndrome [7,11-13].

Unexplained isolated abducens nerve palsies should be investigated with computed tomography and MRI. Classically, 50-70% of patients have lesions accounting for the abducens nerve palsy on MRI [4,11,14], but given the marked improvements in the quality of MRI techniques over the last 10-20 years, culprit lesions are now much more likely to be detected [15].

In patients who present with a clinically isolated syndrome concerning for MS without reasonable historical evidence of a prior attack involving another distinct anatomic location, objective evidence of dissemination of lesions in space and time is required to make a diagnosis of MS per the revised McDonald Criteria of 2017 [16,17]. In our patient, there were FLAIR signal hyperintensities suggestive of active demyelinating lesions in supra- and infra-tentorial areas of the brain (dissemination in space), as well as T1 black holes suggestive of chronic areas of demyelination (dissemination in time). CSF-specific oligoclonal bands may also be used as a surrogate for clinical or radiologic dissemination in time [18].

Once the diagnosis of MS is made following a presentation with a clinically isolated syndrome, patients are started on disease-modifying therapies which have been shown to reduce annual relapse rates [16].

## Conclusions

MS should be considered in the differential diagnosis of bilateral abducens nerve palsy as it can be the initial presenting clinically isolated syndrome in cases of MS. When correlated with a reliable history and radiological findings, this can establish a diagnosis of MS leading to appropriate commencement of the necessary therapeutic measures. Patients who present with bilateral abducens palsy suspicious for MS should undergo fundoscopy and CSF opening pressure measurement to rule out IIH.
